# The Relative Risk of COVID-19 in Solid Organ Transplant Recipients Over Waves of the Pandemic

**DOI:** 10.3389/ti.2024.13351

**Published:** 2024-09-06

**Authors:** Amanda J. Vinson, Alfred J. Anzalone, Makayla Schissel, Ran Dai, Gaurav Agarwal, Stephen B. Lee, Amy Olex, Roslyn B. Mannon

**Affiliations:** ^1^ Division of Nephrology, Department of Medicine, Dalhousie University, Halifax, NS, Canada; ^2^ Department of Biostatistics, University of Nebraska Medical Center, Omaha, NE, United States; ^3^ Division of Nephrology, Department of Medicine, University of Alabama at Birmingham, Birmingham, AL, United States; ^4^ Division of Infectious Diseases (Regina), University of Saskatchewan, Saskatoon, SK, Canada; ^5^ Department of Biostatistics, Virginia Commonwealth University, Virginia, United States; ^6^ Division of Nephology, Department of Medicine, University of Nebraska Medical Center, Omaha, NE, United States

**Keywords:** COVID-19, pandemic, Sars-CoV-2, transplant, outcomes, variant strain, waves, relative risks

## Abstract

Solid organ transplant recipients (SOTR) are at increased risk from COVID-19. Over time, the absolute risk of adverse outcomes after COVID-19 has decreased in both the non-immunosuppressed/immunocompromised (non-ISC) general population, and amongst SOTR. Using the N3C, we examined the absolute risk of mortality, major adverse renal or cardiac events, and hospitalization after COVID-19 diagnosis amongst non-ISC and SOTR populations over five waves of the pandemic (Wave 1: Ancestral COVID; Wave 2: Alpha; Wave 3: Delta; Wave 4: Omicron; Wave 5: Omicron). Within each wave, we determined the relative risk of each outcome for SOTR versus the non-ISC population based on crude event rates, and then used multivariable cox proportional hazards models and logistic regression to determine the adjusted risk of each outcome based on SOT status. Throughout the pandemic, including during the Omicron wave (Wave 5), SOTR were at greater absolute risk for each outcome than non-ISC patients (*p*-values all <0.001). The adjusted risk of SOT status for each outcome was relatively stable over time (aHR 1.28–1.61 for mortality; aHR 1.31–1.47 for MACE; aHR 1.72–1.90 for MARCE; aHR 1.75–2.07 for AKI; and aOR 1.53–1.81 for hospitalization). Despite a reduction in the absolute risk of COVID-19 complications, the relative risk for SOTR versus the non-ISC population has not improved.

## Graphical Abstract

**Figure:**
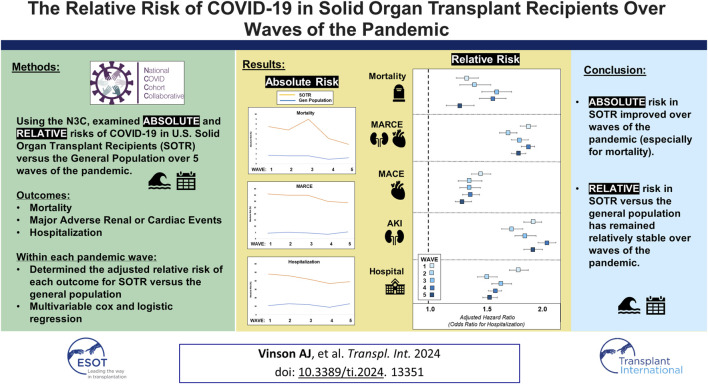

## Introduction

The coronavirus 2019 (COVID-19) pandemic has had dramatic consequences for the population at large, but especially amongst solid organ transplant recipients (SOTR) who are at higher risk for severe infection and mortality [1]. The higher risk in this population is likely on account of exposure to chronic maintenance immunosuppression and greater underlying comorbidity burden [2, 3]. While SOTR benefit from COVID-19 vaccination, vaccine effectiveness among SOTR has been observed to be diminished in comparison to the general population [4]. With the development of effective vaccine programs and more efficacious therapies, COVID-19-related risk of mortality and other complications has improved over time amongst both non-immunosuppressed/immunocompromised (ISC) [5] and SOTR populations [6]. However, whether the *relative* risk associated with SOT, defined as the risk in SOTR compared to that observed in the general population, has improved is unknown. Therefore, in this study we aimed to examine changes in the relative risk of complications post-COVID-19 in SOTR versus non-ISC populations using the largest COVID-19 database in the United States, the National COVID Cohort Collaborative (N3C) [7].

## Methods

The N3C represents a large, national repository of 80 medical centers across the United States contributing data on nearly 9 million adult patients with COVID-19 and more than 14 million COVID-19–negative controls. This centralized and highly granular repository of electronic health record (EHR) data represents the most representative and substantive resource for studying the U.S. COVID-19 population [8]. The N3C includes patients with COVID-19 positivity or suspected positivity by lab testing or diagnostic codes for both inpatient and outpatient encounters [9]. Data is input from four primary data models—OMOP, PCORnet, TriNetX, and ACT—harmonized into the OMOP 5.3.1 data model and made available within a secure enclave for analysis at the patient- and encounter-level [7].

Using the N3C, we examined the absolute risk of 1. mortality (overall), 2. major adverse renal or cardiac events (MARCE; defined as a composite of acute kidney injury (AKI) with or without dialysis, acute myocardial infarction, angina, stent occlusion/thrombosis, stroke, transient ischemic attack, congestive heart failure or death from any cause), 3. major adverse cardiac events (MACE), 4. AKI (defined using condition codes for acute kidney injury or failure) and 5. hospitalization within 90 days of COVID-19 diagnosis amongst non-ISC and SOTR populations (kidney, liver, lung and heart recipients) over five waves of the pandemic (Wave 1: Ancestral COVID; 01/01/2020–12/31/2020; Wave 2: Alpha; 01/01/2021–06/25/2021; Wave 3: Delta; 06/26/2021–12/17/2021; Wave 4: Omicron; 12/18/2021–07/01/2022; Wave 5: Omicron; 07/02/2022–03/31/2023). Outcome events were determined based on diagnostic or procedure codes documented in the 90-day window post-COVID-19 diagnosis and were ascertained using condition codes diagnosed by a provider (e.g., SNOMED CT, ICD-10-CM), procedure codes associated with an encounter within the observation window (CPT4, ICD-10-PCS), or deaths documented within the reporting health system. Individuals without the recorded outcome were assumed to not have the outcome. Patients were considered as belonging to a given wave based on the date of their COVID-19 diagnosis. We also examined the relative risk, comparing the relative risk of each outcome within each wave of the pandemic in SOTR to non-ISC populations. Finally, multivariable Cox proportional hazard models were used to examine the adjusted relative hazard of each outcome associated with SOT status across pandemic waves (multivariable logistic regression for hospitalization at any point within 90 days of COVID diagnosis), with time 0 being date of COVID-19 diagnosis. Models were adjusted for known literature predictors of adverse outcomes after COVID-19 diagnosis, including sex, age, race/ethnicity (White, Black, Hispanic or Latino, Other), comorbidities (chronic kidney disease, hypertension, diabetes, asthma/chronic obstructive pulmonary disease, cancer, peripheral vascular disease, liver disease, obesity, coronary artery disease, congestive heart failure), and vaccination status [no complete vaccination series documented, or breakthrough infection (VAX2: being ≥14 days post two doses for mRNA vaccines, one dose for Johnson & Johnson/Janssen vaccine, or two doses for other vaccines; VAX3: being ≥14 days post a booster dose of any of the above vaccine preparations following VAX2)] [4, 10].

In a secondary analysis, we examined the relative risk of each post-COVID outcome by transplanted organ type (kidney, liver, lung, or heart), rather than for SOTR collectively (based on crude event rates and multivariable modeling as above). Complete case analysis was used for all analyses.

## Results

Among 5.5M non-ISC and 52,630 SOTR with COVID-19, SOTR were significantly older [58 years (Q1 46, Q3 66) versus 45 years (Q1 31, Q3 61)], more likely to be male (58% versus 43%), and with greater comorbidity burden than the general, non-ISC population (73% versus 4.2% with chronic kidney disease; 83% versus 23% with hypertension; 50% versus 11% with diabetes; and 29% versus 3.8% with congestive heart failure), Table 1. SOTR were at significantly higher risk for all outcomes during all waves of the pandemic, Supplementary Figures 1A–E; generally, the absolute risk of each outcome decreased over time for both non-ISC and SOTR. Crude event rates are shown in Supplementary Table 1.

**TABLE 1:** Baseline characteristics at the time of COVID-19 diagnosis in Non-Immunosuppressed/Immunocompromised (Non-ISC) patients and Solid Organ Transplant Recipients (SOTR).

Characteristic	Overall N = 5,521,812	Non-ISC N = 5,469,182	SOTR N = 52,630	*p*-value
Age at COVID-19 Diagnosis	45 (31, 61)	45 (31, 61)	58 (46, 66)	<0.001
Age Strata				<0.001
18–44	2,682,345 (49%)	2,670,289 (49%)	12,056 (23%)	
45–65	1,828,259 (33%)	1,802,261 (33%)	25,998 (49%)	
>65	1,011,208 (18%)	996,632 (18%)	14,576 (28%)	
Sex				<0.001
Female	3,136,019 (57%)	3,113,865 (57%)	22,154 (42%)	
Male	2,385,793 (43%)	2,355,317 (43%)	30,476 (58%)	
Race/Ethnicity				<0.001
White	3,425,575 (62%)	3,397,683 (62%)	27,892 (53%)	
Black or African American	683,952 (12%)	672,713 (12%)	11,239 (21%)	
Hispanic or Latino	670,690 (12%)	662,772 (12%)	7,918 (15%)	
Other/Unknown	741,595 (13%)	736,014 (13%)	5,581 (11%)	
Comorbidities				
CKD	268,409 (4.9%)	229,872 (4.2%)	38,537 (73%)	<0.001
Hypertension	1,318,062 (24%)	1,274,263 (23%)	43,799 (83%)	<0.001
Diabetes	635,247 (12%)	609,175 (11%)	26,072 (50%)	<0.001
COPD/Asthma	549,062 (9.9%)	539,611 (9.9%)	9,451 (18%)	<0.001
Cancer	315,939 (5.7%)	305,293 (5.6%)	10,646 (20%)	<0.001
CAD	293,049 (5.3%)	278,109 (5.1%)	14,940 (28%)	<0.001
CHF	221,868 (4.0%)	206,819 (3.8%)	15,049 (29%)	<0.001
PVD	236,408 (4.3%)	224,633 (4.1%)	11,775 (22%)	<0.001
Liver Disease	213,042 (3.9%)	200,494 (3.7%)	12,548 (24%)	<0.001
Obesity	1,723,831 (31%)	1,695,716 (31%)	28,115 (53%)	<0.001
Vaccination Status				<0.001
Non-Breakthrough Infection	4,612,447 (84%)	4,571,722 (84%)	40,725 (77%)	
VAX2 Breakthrough Infection	531,876 (9.6%)	526,492 (9.6%)	5,384 (10%)	
VAX3 Breakthrough Infection	377,489 (6.8%)	370,968 (6.8%)	6,521 (12%)	
SARS-CoV-2 Variant Wave				<0.001
Ancestral COVID-19	1,492,240 (27%)	1,481,743 (27%)	10,497 (20%)	
Alpha (B.1.1.7), Beta (B.1.351), Gamma (P.1)	655,400 (12%)	649,065 (12%)	6,335 (12%)	
Delta (B.1.617.2)	926,564 (17%)	918,803 (17%)	7,761 (15%)	
Omicron (B.1.1.529, BA.2, BA.2.12.1)	1,520,782 (28%)	1,504,310 (28%)	16,472 (31%)	
Omicron (BA.5, BQ.1.1, XBB.1.5)	926,826 (17%)	915,261 (17%)	11,565 (22%)	
Transplantation Type				N/A
Non-Transplant	5,469,182 (99%)	5,469,182 (100%)	0	
Kidney	33,412 (0.6%)	N/A	33,412 (63%)	
Liver	8,545 (0.2%)	N/A	8,545 (16%)	
Lung	4,883 (<0.1%)	N/A	4,883 (9.3%)	
Heart	5,790 (0.1%)	N/A	5,790 (11%)	

**FIGURE 1:**
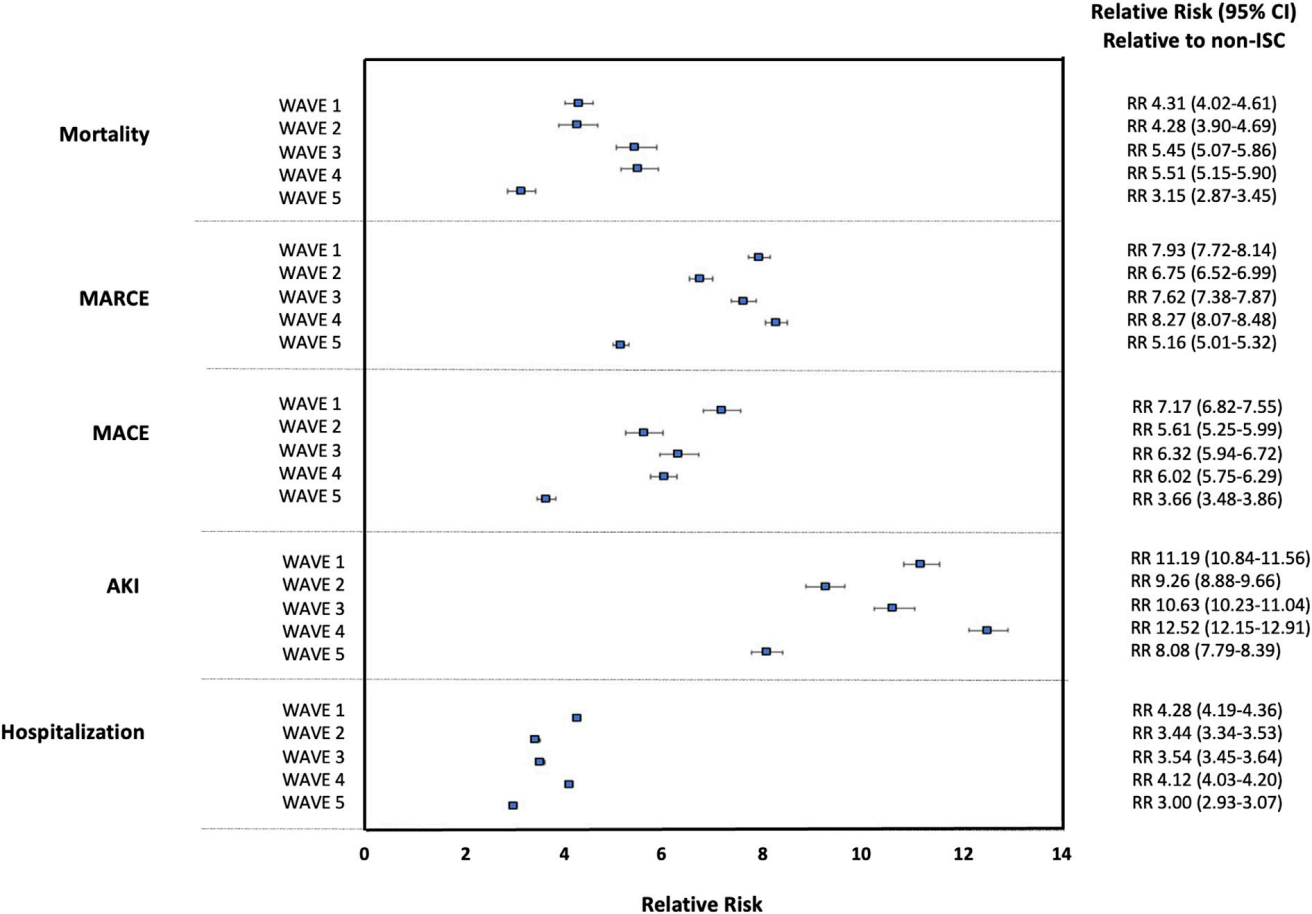
Relative Risk for 90 days Outcomes Post-COVID-19 for Solid Organ Transplant Recipients (SOTR) Versus non-Immunosuppressed/Immunocompromised (non-ISC) Populations Over Waves of the Pandemic.

The relative risk (SOTR versus non-ISC) based on crude event rates for each outcome over waves of the pandemic is shown in Figure 1. Throughout the pandemic, including during the Omicron wave (Wave 5), SOTR were at ∼3-8x greater risk for each outcome than non-ISC patients and ∼8-12x greater risk for AKI. Compared with the general population, the relative risk for SOTR was greatest during Wave 1 (for the outcomes of MACE and hospitalization) and Wave 4 (for the outcomes of mortality, MARCE, and AKI). The relative risk in SOTR versus the general population was significantly lower in Wave 5 for each outcome.

**FIGURE 2:**
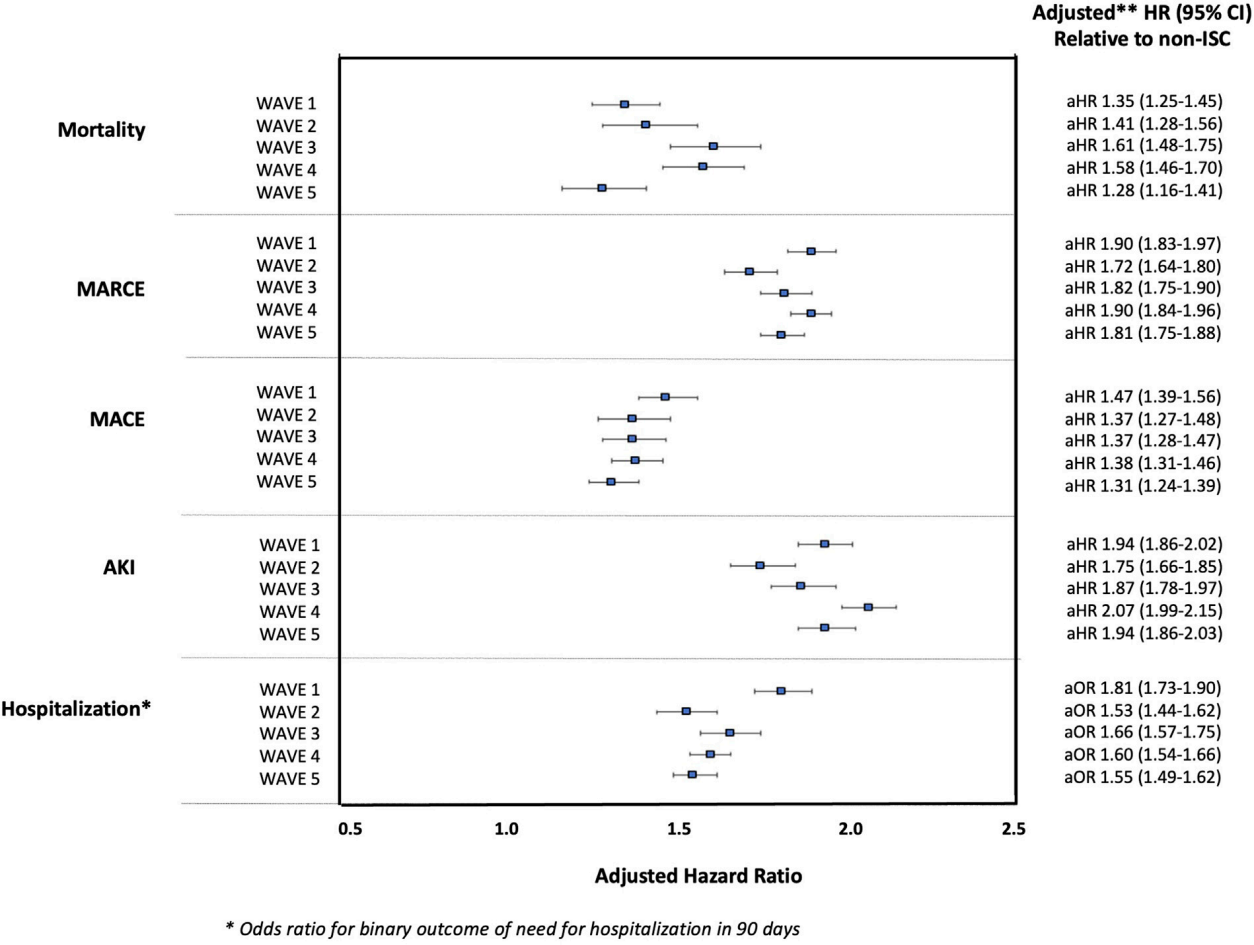
Adjusted Hazard Ratios for 90-day Outcomes Post-COVID-19 (and Odds Ratio for Hospitalization) in SOTR Versus non-ISC Populations Over Waves of the Pandemic.

In multivariable models, SOT status was significantly associated with increased risk for each outcome during all waves of the pandemic (*p*-values all <0.001). The adjusted risk of SOT status for each outcome was relatively stable over time (aHR 1.28–1.61 for mortality; aHR 1.31–1.47 for MACE; aHR 1.72–1.90 for MARCE; aHR 1.75–2.07 for AKI; and aOR 1.53–1.81 for hospitalization), Figure 2. The adjusted risk of each outcome associated with SOT status (relative to the general, non-ISC population) over the five waves of the pandemic is shown in Supplementary Table 2. The adjusted risk for SOTR versus non-ISC was highest in Wave 1 (for the outcomes of MARCE, MACE and hospitalization) and Wave 4 (for the outcomes of mortality and AKI), though there was substantial overlap in confidence intervals with no significant differences noted in SOT-associated risk across the waves as a whole. In adjusted models, SOTR were not at significantly lower risk for any outcome during Wave 5 compared with other waves.

Finally, the relative risk for each outcome by organ type based on crude event rates is shown in Supplementary Table 3, and the adjusted relative risk by organ type based on multivariable modeling is shown in Supplementary Table 4. Relative to non-ISC, the highest mortality risk was for lung transplant recipients [aHR 1.93, 95% CI 1.53–2.43 in Wave 3 (minimum); aHR 2.27, 95% CI 1.86–2.76 in Wave 4 (maximum)], followed by kidney transplant recipients [aHR 1.46, 95% CI 1.28–1.66 in Wave 5 (minimum); aHR 1.84, 95% CI 1.68–2.01 in Wave 4 (maximum)].

## Discussion

Although the absolute risk associated with SARS-CoV-2 infection has decreased over time in both SOTR and the general population, SOTR remain at significantly higher risk for complications and serious adverse events post-COVID-19 than the general, non-ISC population; this has not improved over time after adjusting for potential confounders. In keeping with earlier literature [11–13], relative to the general population, the risk of death was highest amongst lung, followed by kidney transplant recipients, though the organ-specific risk across all waves of the pandemic was overall stable.

Since the onset of the pandemic, SOTR have experienced disproportionately higher rates of COVID-19 complications (including greater case-fatality ratios) than the general population, which has been attributed largely to their state of chronic immunosuppression and increased baseline comorbidity burden [1]. Relatively widespread uptake of efficacious vaccination programs and improved anti-SARS-CoV-2 therapeutic strategies have reduced the overall risk of serious adverse events post-COVID-19 [5]. The slight downtrend in relative risk for SOTR during Wave 2 may reflect prioritized access to vaccination for immunosuppressed patients during this period.

Importantly, although COVID-19 risk has diminished over time, there remains a substantial burden of illness attributable to SARS-CoV-2 infection with hospitalization rates for SOTR and non-ISC of 39.3% and 13.1% amongst those with a positive COVID-19 result recorded in the N3C during Wave 5. While a limitation of the data is that home COVID-19 testing results are not captured, these patients would typically be more likely to have asymptomatic or mild disease, and the absolute number (not percentage) of patients with each complication post-COVID would be unlikely to change (n = 4,548 and n = 120,010 hospitalizations amongst SOTR and non-ISC during Wave 5). Notably however, if asymptomatic and mild cases were completely captured, the proportion of patients requiring hospitalization after a positive test (not the absolute number) would likely be smaller. It is also important to note that the time at risk for COVID infection (duration of an individual wave) was not consistent across waves, as the dates were chosen to reflect a dominant circulating variant strain rather than a given period of time [e.g., Wave 1 (Ancestral) lasted 12 months whereas Wave 3 (Delta) lasted less than 6 months]. Therefore, comparison of isolated absolute event rates (rather than relative rates) across pandemic periods is not possible. Hence, the multivariable models we conducted (displayed in Figure 2), are the most accurate representation of relative risk associated with SOT status given the above limitations. An additional limitation of the current study is that within each variant period, we cannot comment on changing trends over time, rather we present the overall absolute and relative risks for SOTR versus non-ISC populations in a given time period, acknowledging the potential for change in risk over a given wave.

Overall, there has been a reduction in the absolute risk of COVID-19 complications amongst both SOTR and non-ISC populations over the pandemic. However, risk is not negligible, and SOTR remain at significantly higher risk than the general population; SOTR continue to be disproportionately impacted by COVID-19.

## Data Availability

The N3C Enclave is available for public research use. To access data, institutions must have a signed Data Use Agreement executed with the US National Center for Advancing Translational Sciences (NCATS), and investigators must complete mandatory training and submit a Data Use Request (DUR) to N3C. To request N3C data access, follow the instructions at https://covid.cd2h.org/onboarding. All code used for analyses can be made available upon request. More than 4000 researchers currently have access to data in N3C, representing more than 300 US research institution. Details are provided in the supplement.

## Group Members of National COVID Cohort Collaborative (N3C) Consortium

Adam B. Wilcox, Adam M. Lee, Alexis Graves, Alfred (Jerrod) Anzalone, Amin Manna, Amit Saha, Amy Olex, Andrea Zhou, Andrew E. Williams, Andrew Southerland, Andrew T. Girvin, Anita Walden, Anjali A. Sharathkumar, Benjamin Amor, Benjamin Bates, Brian Hendricks, Brijesh Patel, Caleb Alexander, Carolyn Bramante, Cavin Ward-Caviness, Charisse Madlock-Brown, Christine Suver, Christopher Chute, Christopher Dillon, Chunlei Wu, Clare Schmitt, Cliff Takemoto, Dan Housman, Davera Gabriel, David A. Eichmann, Diego Mazzotti, Don Brown, Eilis Boudreau, Elaine Hill, Elizabeth Zampino, Emily Carlson Marti, Emily R. Pfaff, Evan French, Farrukh M. Koraishy, Federico Mariona, Fred Prior, George Sokos, Greg Martin, Harold Lehmann, Heidi Spratt, Hemalkumar Mehta, Hongfang Liu, Hythem Sidky, J. W. Awori Hayanga, Jami Pincavitch, Jaylyn Clark, Jeremy Richard Harper, Jessica Islam, Jin Ge, Joel Gagnier, Joel H. Saltz, Joel Saltz, Johanna Loomba, John Buse, Jomol Mathew, Joni L. Rutter, Julie A. McMurry, Justin Guinney, Justin Starren, Karen Crowley, Katie Rebecca Bradwell, Kellie M. Walters, Ken Wilkins, Kenneth R. Gersing, Kenrick Dwain Cato, Kimberly Murray, Kristin Kostka, Lavance Northington, Lee Allan Pyles, Leonie Misquitta, Lesley Cottrell, Lili Portilla, Mariam Deacy, Mark M. Bissell, Marshall Clark, Mary Emmett, Mary Morrison Saltz, Matvey B. Palchuk, Melissa A. Haendel, Meredith Adams, Meredith Temple-O’Connor, Michael G. Kurilla, Michele Morris, Nabeel Qureshi, Nasia Safdar, Nicole Garbarini, Noha Sharafeldin, Ofer Sadan, Patricia A. Francis, Penny Wung Burgoon, Peter Robinson, Philip R. O. Payne, Rafael Fuentes, Randeep Jawa, Rebecca Erwin-Cohen, Rena Patel, Richard A. Moffitt, Richard L. Zhu, Rishi Kamaleswaran, Robert Hurley, Robert T. Miller, Saiju Pyarajan, Sam G. Michael, Samuel Bozzette, Sandeep Mallipattu, Satyanarayana Vedula, Scott Chapman, Shawn T. O’Neil, Soko Setoguchi, Stephanie S. Hong, Steve Johnson, Tellen D. Bennett, Tiffany Callahan, Umit Topaloglu, Usman Sheikh, Valery Gordon, Vignesh Subbian, Warren A. Kibbe, Wenndy Hernandez, Will Beasley, Will Cooper, William Hillegass, Xiaohan Tanner Zhang. Details of contributions available at http://covid.cd2h.org/core-contributors.
