# Supplementary material for: The Relative Risk of COVID-19 in Solid Organ Transplant Recipients Over Waves of the Pandemic
Source: Transpl Int. 2024 Sep 6;37:13351. doi: 10.3389/ti.2024.13351 (PMC11413870; doi:10.3389/ti.2024.13351)
Supplement: Supplementary file 2 [file DataSheet1.docx]

**Supplement:**

**Supplemental Table 1: Crude Event Rates For Non-Immunosuppressed/Immunocompromised and Solid Organ Transplant Populations Over Waves of the Pandemic.**

| **Characteristic** | **Wave 1**  **Ancestral**  **COVID-19** | **Wave 2**  **Alpha (B.1.1.7),**  **Beta (B.1.351),**  **Gamma (P.1)** | **Wave 3**  **Delta**  **(B.1.617.2)** | **Wave 4**  **Omicron (B.1.1.529, BA.2, BA.2.12.1)** | **Wave 5**  **Omicron**  **(BA.5, BQ.1.1, XBB.1.5)** |
| --- | --- | --- | --- | --- | --- |
| **Mortality** | | | | | |
| Non-ISC | 25,736 / 1,481,743  (1.7%) | 10,327 / 649,065 (1.6%) | 15,127 / 918,803 (1.6%) | 13,811 / 1,504,310 (0.92%) | 11,294 / 915,261 (1.2%) |
| SOT | 785 / 10,497  (7.5%) | 431 / 6,335  (6.8%) | 696 / 7,761  (9.0%) | 834 / 16,472  (5.1%) | 449 / 11,565 (3.9%) |
| **MARCE** | | | | | |
| Non-ISC | 67,034 / 1,481,743 (4.5%) | 33,671 / 649,065 (5.2%) | 41,910 / 918,803 (4.6%) | 53,706 / 1,504,310 (3.6%) | 50,600 / 915,261 (5.5%) |
| SOT | 3,765 / 10,497 (36%) | 2,218 / 6,335  (35%) | 2,697 / 7,761  (35%) | 4,866 / 16,472 (30%) | 3,301 / 11,565 (29%) |
| **MACE** | | | | | |
| Non-ISC | 27,117 / 1,481,743 (1.8%) | 15,097 / 649,065 (2.3%) | 17,442 / 918,803 (1.9%) | 27,701 / 1,504,310 (1.8%) | 28,179 / 915,261 (3.1%) |
| SOT | 1,378 / 10,497 (13%) | 826 / 6,335  (13%) | 931 / 7,761  (12%) | 1,825 / 16,472 (11%) | 1,304 / 11,565 (11%) |
| **AKI** | | | | | |
| Non-ISC | 37,242 / 1,481,743 (2.5%) | 19,240 / 649,065 (3.0%) | 23,744 / 918,803 (2.6%) | 27,504 / 1,504,310 (1.8%) | 24,253 / 915,261 (2.6%) |
| SOT | 2,953 / 10,497 (28%) | 1,739 / 6,335  (27%) | 2,132 / 7,761  (27%) | 3,772 / 16,472 (23%) | 2,477 / 11,565 (21%) |
| **Hospitalization** | | | | | |
| Non-ISC | 167,520 / 1,481,743 (11%) | 86,809 / 649,065 (13%) | 108,909 / 918,803 (12%) | 134,694 / 1,504,310 (9.0%) | 120,010 / 915,261 (13%) |
| SOT | 5,074 / 10,497 (48%) | 2,912 / 6,335  (46%) | 3,260 / 7,761  (42%) | 6,075 / 16,472 (37%) | 4,548 / 11,565 (39%) |

**Supplemental Table 2. Multivariable Cox and Logistic Regression for Post-COVID-19 Events Across Variant Waves by Solid Organ Transplantation Status**

1. **All Time Periods**

| **Characteristic** | **Death, aHR (95% CI)** | **MACE, aHR (95% CI)** | **MARCE, aHR (95% CI)** | **AKI/Dialysis, aHR (95% CI)** | **Hospitalization, aOR (95% CI)** |
| --- | --- | --- | --- | --- | --- |
| Sex |  |  |  |  |  |
| Female | Reference | Reference | Reference | Reference | Reference |
| Male | 1.58 (1.55, 1.60) | 1.47 (1.46, 1.49) | 1.56 (1.55, 1.57) | 1.64 (1.62, 1.66) | 1.08 (1.07, 1.08) |
| Age at COVID-19 Diagnosis | 1.07 (1.07, 1.07) | 1.05 (1.05, 1.05) | 1.05 (1.05, 1.05) | 1.04 (1.04, 1.04) | 1.03 (1.02, 1.03) |
| Race/Ethnicity |  |  |  |  |  |
| White | Reference | Reference | Reference | Reference | Reference |
| Black or African American | 1.30 (1.27, 1.33) | 1.60 (1.58, 1.63) | 1.83 (1.81, 1.85) | 2.17 (2.14, 2.20) | 1.63 (1.62, 1.64) |
| Hispanic or Latino | 1.26 (1.23, 1.29) | 1.07 (1.05, 1.10) | 1.26 (1.24, 1.28) | 1.50 (1.47, 1.52) | 1.59 (1.57, 1.60) |
| Other/Unknown | 1.19 (1.17, 1.22) | 1.05 (1.03, 1.07) | 1.13 (1.12, 1.15) | 1.26 (1.24, 1.29) | 0.98 (0.97, 0.99) |
| SOT Status |  |  |  |  |  |
| Non-SOT Recipient | Reference | Reference | Reference | Reference | Reference |
| SOT Recipient | 1.45 (1.40, 1.51) | 1.38 (1.35, 1.42) | 1.85 (1.82, 1.88) | 1.94 (1.90, 1.98) | 1.66 (1.63, 1.69) |
| CKD |  |  |  |  |  |
| No History of CKD | Reference | Reference | Reference | Reference | Reference |
| History of CKD | 1.55 (1.52, 1.58) | 2.04 (2.02, 2.07) | 2.34 (2.32, 2.37) | 2.90 (2.86, 2.94) | 1.67 (1.66, 1.69) |
| Hypertension |  |  |  |  |  |
| No History of Hypertension | Reference | Reference | Reference | Reference | Reference |
| History of Hypertension | 1.26 (1.23, 1.28) | 3.09 (3.04, 3.14) | 2.29 (2.27, 2.32) | 2.41 (2.37, 2.44) | 1.37 (1.36, 1.38) |
| Diabetes |  |  |  |  |  |
| No History of Diabetes | Reference | Reference | Reference | Reference | Reference |
| History of Diabetes | 1.39 (1.37, 1.41) | 1.52 (1.50, 1.54) | 1.54 (1.52, 1.55) | 1.58 (1.56, 1.60) | 1.58 (1.56, 1.59) |
| Asthma |  |  |  |  |  |
| No History of Asthma | Reference | Reference | Reference | Reference | Reference |
| History of Asthma | 1.27 (1.25, 1.30) | 1.73 (1.71, 1.75) | 1.46 (1.45, 1.48) | 1.16 (1.14, 1.17) | 1.46 (1.45, 1.48) |
| Cancer |  |  |  |  |  |
| No History of Cancer | Reference | Reference | Reference | Reference | Reference |
| History of Cancer | 1.42 (1.40, 1.45) | 0.88 (0.87, 0.90) | 1.05 (1.04, 1.06) | 1.0 (0.98, 1.01) | 1.32 (1.31, 1.33) |
| PVD |  |  |  |  |  |
| No History of PVD | Reference | Reference | Reference | Reference | Reference |
| History of PVD | 1.06 (1.04, 1.08) | 1.35 (1.33, 1.37) | 1.18 (1.17, 1.19) | 0.97 (0.96, 0.99) | 1.20 (1.19, 1.22) |
| Liver Disease |  |  |  |  |  |
| No History of Liver Disease | Reference | Reference | Reference | Reference | Reference |
| History of Liver Disease | 2.07 (2.03, 2.12) | 1.41 (1.39, 1.44) | 1.56 (1.54, 1.58) | 1.67 (1.65, 1.70) | 1.64 (1.63, 1.66) |
| Obesity |  |  |  |  |  |
| No History of Obesity | Reference | Reference | Reference | Reference | Reference |
| History of Obesity | 1.13 (1.11, 1.14) | 1.31 (1.29, 1.33) | 1.19 (1.19, 1.21) | 1.13 (1.12, 1.15) | 1.27 (1.27, 1.28) |
| CAD |  |  |  |  |  |
| No History of CAD | Reference | N/A | N/A | Reference | Reference |
| History of CAD | 0.97 (0.96, 0.99) | N/A | N/A | 0.98 (0.97, 0.99) | 1.19 (1.18, 1.21) |
| CHF |  |  |  |  |  |
| No History of CHF | Reference | N/A | N/A | Reference | Reference |
| History of CHF | 1.93 (1.89, 1.97) | N/A | N/A | 1.75 (1.72, 1.77) | 2.21 (2.19, 2.24) |
| Vaccination Status Before COVID-19 |  |  |  |  |  |
| Non-Breakthrough Infection | Reference | Reference | Reference | Reference | Reference |
| VAX2 Breakthrough Infection | 0.45 (0.44, 0.47) | 0.61 (0.60, 0.62) | 0.54 (0.53, 0.55) | 0.48 (0.47, 0.49) | 0.53 (0.52, 0.53) |
| VAX3 Breakthrough Infection | 0.42 (0.41, 0.44) | 0.50 (0.49, 0.51) | 0.46 (0.45, 0.47) | 0.41 (0.40, 0.42) | 0.40 (0.40, 0.41) |
| Variant Wave |  |  |  |  |  |
| Alpha (B.1.1.7), Beta (B.1.351), Gamma (P.1) | Reference | Reference | Reference | Reference | Reference |
| Ancestral COVID-19 | 1.13 (1.11, 1.16) | 0.85 (0.84, 0.87) | 0.93 (0.92, 0.94) | 0.92 (0.90, 0.93) | 0.86 (0.85, 0.87) |
| Delta (B.1.617.2) | 1.35 (1.31, 1.38) | 1.00 (0.98, 1.02) | 1.09 (1.08, 1.11) | 1.14 (1.12, 1.16) | 1.02 (1.01, 1.03) |
| Omicron (B.1.1.529, BA.2, BA.2.12.1) | 0.68 (0.66, 0.69) | 0.88 (0.86, 0.90) | 0.78 (0.77, 0.79) | 0.75 (0.74, 0.77) | 0.72 (0.72, 0.73) |
| Omicron (BA.5, BQ.1.1, XBB.1.5) | 0.53 (0.51, 0.54) | 0.90 (0.88, 0.92) | 0.76 (0.75, 0.77) | 0.71 (0.70, 0.73) | 0.85 (0.84, 0.86) |

1. **Wave 1: Ancestral COVID-19**

| **Characteristic** | **Death,**  **aHR (95% CI)** | **MACE,**  **aHR (95% CI)** | **MARCE,**  **aHR (95% CI)** | **AKI/Dialysis,**  **aHR (95% CI)** | **Hospitalization,**  **aOR (95% CI)** |
| --- | --- | --- | --- | --- | --- |
| Sex |  |  |  |  |  |
| Female | Reference | Reference | Reference | Reference | Reference |
| Male | 1.70 (1.66, 1.74) | 1.50 (1.47, 1.54) | 1.65 (1.62, 1.67) | 1.78 (1.75, 1.82) | 1.20 (1.18, 1.21) |
| Age at COVID-19 Diagnosis | 1.08 (1.08, 1.08) | 1.05 (1.05, 1.05) | 1.06 (1.06, 1.06) | 1.05 (1.04, 1.05) | 1.04 (1.04, 1.04) |
| Race/Ethnicity |  |  |  |  |  |
| White | Reference | Reference | Reference | Reference | Reference |
| Black or African American | 1.39 (1.34, 1.44) | 1.75 (1.70, 1.80) | 2.03 (1.99, 2.07) | 2.54 (2.48, 2.60) | 1.84 (1.81, 1.87) |
| Hispanic or Latino | 1.46 (1.41, 1.52) | 1.21 (1.17, 1.26) | 1.47 (1.44, 1.51) | 1.81 (1.76, 1.86) | 1.70 (1.68, 1.73) |
| Other/Unknown | 1.26 (1.22, 1.31) | 1.11 (1.07, 1.16) | 1.25 (1.22, 1.29) | 1.48 (1.44, 1.53) | 1.11 (1.09, 1.13) |
| SOT Status |  |  |  |  |  |
| Non-SOT Recipient | Reference | Reference | Reference | Reference | Reference |
| SOT Recipient | 1.35 (1.25, 1.45) | 1.47 (1.39, 1.56) | 1.90 (1.83, 1.97) | 1.94 (1.86, 2.02) | 1.81 (1.73, 1.90) |
| CKD |  |  |  |  |  |
| No History of CKD | Reference | Reference | Reference | Reference | Reference |
| History of CKD | 1.58 (1.53, 1.63) | 2.09 (2.03, 2.15) | 2.28 (2.24, 2.32) | 2.71 (2.64, 2.78) | 1.78 (1.74, 1.82) |
| Hypertension |  |  |  |  |  |
| No History of Hypertension | Reference | Reference | Reference | Reference | Reference |
| History of Hypertension | 1.33 (1.29, 1.38) | 3.29 (3.18, 3.41) | 2.30 (2.26, 2.35) | 2.61 (2.54, 2.69) | 1.43 (1.41, 1.45) |
| Diabetes |  |  |  |  |  |
| No History of Diabetes | Reference | Reference | Reference | Reference | Reference |
| History of Diabetes | 1.44 (1.40, 1.48) | 1.57 (1.52, 1.61) | 1.59 (1.56, 1.62) | 1.66 (1.62, 1.70) | 1.71 (1.69, 1.74) |
| Asthma |  |  |  |  |  |
| No History of Asthma | Reference | Reference | Reference | Reference | Reference |
| History of Asthma | 1.32 (1.28, 1.36) | 1.73 (1.68, 1.77) | 1.44 (1.42, 1.47) | 1.17 (1.14, 1.20) | 1.60 (1.57, 1.63) |
| Cancer |  |  |  |  |  |
| No History of Cancer | Reference | Reference | Reference | Reference | Reference |
| History of Cancer | 1.23 (1.19, 1.27) | 0.90 (0.87, 0.93) | 1.02 (1.00, 1.04) | 0.95 (0.92, 0.97) | 1.30 (1.27, 1.32) |
| PVD |  |  |  |  |  |
| No History of PVD | Reference | Reference | Reference | Reference | Reference |
| History of PVD | 1.03 (0.99, 1.07) | 1.31 (1.27, 1.35) | 1.10 (1.08, 1.13) | 0.92 (0.89, 0.95) | 1.18 (1.15, 1.21) |
| Liver Disease |  |  |  |  |  |
| No History of Liver Disease | Reference | Reference | Reference | Reference | Reference |
| History of Liver Disease | 2.00 (1.92, 2.09) | 1.48 (1.43, 1.54) | 1.58 (1.54, 1.62) | 1.64 (1.59, 1.69) | 1.71 (1.67, 1.75) |
| Obesity |  |  |  |  |  |
| No History of Obesity | Reference | Reference | Reference | Reference | Reference |
| History of Obesity | 1.25 (1.22, 1.28) | 1.41 (1.37, 1.44) | 1.30 (1.28, 1.32) | 1.26 (1.23, 1.29) | 1.53 (1.51, 1.55) |
| CAD |  |  |  |  |  |
| No History of CAD | Reference | N/A | N/A | Reference | Reference |
| History of CAD | 1.01 (0.98, 1.05) | N/A | N/A | 0.98 (0.96, 1.01) | 1.19 (1.16, 1.21) |
| CHF |  |  |  |  |  |
| No History of CHF | Reference | N/A | N/A | Reference | Reference |
| History of CHF | 1.66 (1.61, 1.72) | N/A | N/A | 1.48 (1.44, 1.52) | 1.95 (1.90, 1.99) |

1. **Wave 2: Alpha (B.1.1.7), Beta (B.1.351), Gamma (P.1)**

| **Characteristic** | **Death,**  **aHR (95% CI)** | **MACE,**  **aHR (95% CI)** | **MARCE,**  **aHR (95% CI)** | **AKI/Dialysis,**  **aHR (95% CI)** | **Hospitalization,**  **aOR (95% CI)** |
| --- | --- | --- | --- | --- | --- |
| Sex |  |  |  |  |  |
| Female | Reference | Reference | Reference | Reference | Reference |
| Male | 1.59 (1.53, 1.65) | 1.53 (1.48, 1.58) | 1.59 (1.56, 1.63) | 1.71 (1.66, 1.75) | 1.14 (1.12, 1.15) |
| Age at COVID-19 Diagnosis | 1.07 (1.07, 1.07) | 1.05 (1.05, 1.05) | 1.05 (1.05, 1.05) | 1.04 (1.04, 1.04) | 1.03 (1.03, 1.03) |
| Race/Ethnicity |  |  |  |  |  |
| White | Reference | Reference | Reference | Reference | Reference |
| Black or African American | 1.34 (1.27, 1.41) | 1.59 (1.53, 1.65) | 1.88 (1.83, 1.93) | 2.26 (2.19, 2.34) | 1.76 (1.73, 1.80) |
| Hispanic or Latino | 1.28 (1.20, 1.36) | 1.02 (0.96, 1.07) | 1.23 (1.19, 1.27) | 1.45 (1.39, 1.52) | 1.62 (1.59, 1.66) |
| Other/Unknown | 1.30 (1.22, 1.38) | 1.12 (1.06, 1.18) | 1.25 (1.20, 1.29) | 1.41 (1.34, 1.47) | 1.14 (1.11, 1.17) |
| SOT Status |  |  |  |  |  |
| Non-SOT Recipient | Reference | Reference | Reference | Reference | Reference |
| SOT Recipient | 1.41 (1.28, 1.56) | 1.37 (1.27, 1.48) | 1.72 (1.64, 1.80) | 1.75 (1.66, 1.85) | 1.53 (1.44, 1.62) |
| CKD |  |  |  |  |  |
| No History of CKD | Reference | Reference | Reference | Reference | Reference |
| History of CKD | 1.55 (1.47, 1.63) | 2.03 (1.96, 2.11) | 2.38 (2.32, 2.44) | 2.83 (2.73, 2.93) | 1.71 (1.66, 1.76) |
| Hypertension |  |  |  |  |  |
| No History of Hypertension | Reference | Reference | Reference | Reference | Reference |
| History of Hypertension | 1.19 (1.13, 1.25) | 2.99 (2.86, 3.13) | 2.21 (2.15, 2.27) | 2.30 (2.21, 2.39) | 1.37 (1.34, 1.39) |
| Diabetes |  |  |  |  |  |
| No History of Diabetes | Reference | Reference | Reference | Reference | Reference |
| History of Diabetes | 1.37 (1.31, 1.44) | 1.51 (1.46, 1.57) | 1.52 (1.48, 1.55) | 1.59 (1.54, 1.64) | 1.53 (1.49, 1.56) |
| Asthma |  |  |  |  |  |
| No History of Asthma | Reference | Reference | Reference | Reference | Reference |
| History of Asthma | 1.19 (1.13, 1.25) | 1.68 (1.62, 1.74) | 1.39 (1.35, 1.42) | 1.13 (1.09, 1.17) | 1.48 (1.44, 1.51) |
| Cancer |  |  |  |  |  |
| No History of Cancer | Reference | Reference | Reference | Reference | Reference |
| History of Cancer | 1.36 (1.29, 1.43) | 0.88 (0.84, 0.93) | 1.05 (1.02, 1.08) | 1.00 (0.96, 1.04) | 1.32 (1.28, 1.36) |
| PVD |  |  |  |  |  |
| No History of PVD | Reference | Reference | Reference | Reference | Reference |
| History of PVD | 1.08 (1.02, 1.14) | 1.33 (1.27, 1.38) | 1.15 (1.12, 1.18) | 0.97 (0.93, 1.01) | 1.23 (1.19, 1.27) |
| Liver Disease |  |  |  |  |  |
| No History of Liver Disease | Reference | Reference | Reference | Reference | Reference |
| History of Liver Disease | 2.14 (2.02, 2.27) | 1.38 (1.31, 1.45) | 1.54 (1.48, 1.59) | 1.70 (1.63, 1.77) | 1.67 (1.62, 1.73) |
| Obesity |  |  |  |  |  |
| No History of Obesity | Reference | Reference | Reference | Reference | Reference |
| History of Obesity | 1.20 (1.15, 1.25) | 1.38 (1.33, 1.43) | 1.27 (1.24, 1.30) | 1.22 (1.18, 1.25) | 1.52 (1.49, 1.54) |
| CAD |  |  |  |  |  |
| No History of CAD | Reference | N/A | N/A | Reference | Reference |
| History of CAD | 1.00 (0.95, 1.06) | N/A | N/A | 0.95 (0.91, 0.98) | 1.18 (1.15, 1.22) |
| CHF |  |  |  |  |  |
| No History of CHF | Reference | N/A | N/A | Reference | Reference |
| History of CHF | 1.83 (1.74, 1.92) | N/A | N/A | 1.64 (1.58, 1.70) | 2.09 (2.02, 2.15) |
| Vaccination Status Before COVID-19 | | |  |  |  |
| Non-Breakthrough Infection | Reference | Reference | Reference | Reference | Reference |
| VAX2 Breakthrough Infection | 0.36 (0.29, 0.44) | 0.73 (0.65, 0.83) | 0.57 (0.52, 0.62) | 0.47 (0.41, 0.54) | 0.62 (0.58, 0.67) |

1. **Wave 3: Delta (B.1.617.2)**

| **Characteristic** | **Death,**  **aHR (95% CI)** | **MACE,**  **aHR (95% CI)** | **MARCE,**  **aHR (95% CI)** | **AKI/Dialysis,**  **aHR (95% CI)** | **Hospitalization,**  **aOR (95% CI)** |
| --- | --- | --- | --- | --- | --- |
| Sex |  |  |  |  |  |
| Female | Reference | Reference | Reference | Reference | Reference |
| Male | 1.62 (1.57, 1.67) | 1.49 (1.45, 1.53) | 1.62 (1.59, 1.65) | 1.74 (1.70, 1.79) | 1.15 (1.13, 1.17) |
| Age at COVID-19 Diagnosis | 1.06 (1.06, 1.07) | 1.05 (1.05, 1.05) | 1.05 (1.05, 1.05) | 1.04 (1.04, 1.04) | 1.03 (1.03, 1.03) |
| Race/Ethnicity |  |  |  |  |  |
| White | Reference | Reference | Reference | Reference | Reference |
| Black or African American | 1.21 (1.16, 1.27) | 1.74 (1.67, 1.81) | 1.96 (1.91, 2.01) | 2.31 (2.24, 2.38) | 1.62 (1.59, 1.65) |
| Hispanic or Latino | 1.31 (1.24, 1.40) | 1.17 (1.10, 1.24) | 1.35 (1.30, 1.40) | 1.53 (1.46, 1.60) | 1.62 (1.58, 1.65) |
| Other/Unknown | 1.39 (1.32, 1.46) | 1.22 (1.16, 1.29) | 1.30 (1.25, 1.34) | 1.35 (1.29, 1.41) | 1.02 (0.99, 1.04) |
| SOT Status |  |  |  |  |  |
| Non-SOT Recipient | Reference | Reference | Reference | Reference | Reference |
| SOT Recipient | 1.61 (1.48, 1.75) | 1.37 (1.28, 1.47) | 1.82 (1.75, 1.90) | 1.87 (1.78, 1.97) | 1.66 (1.57, 1.75) |
| CKD |  |  |  |  |  |
| No History of CKD | Reference | Reference | Reference | Reference | Reference |
| History of CKD | 1.51 (1.44, 1.58) | 2.00 (1.93, 2.08) | 2.27 (2.22, 2.33) | 2.77 (2.68, 2.86) | 1.62 (1.58, 1.66) |
| Hypertension |  |  |  |  |  |
| No History of Hypertension | Reference | Reference | Reference | Reference | Reference |
| History of Hypertension | 1.30 (1.25, 1.36) | 3.11 (2.98, 3.24) | 2.18 (2.12, 2.23) | 2.24 (2.16, 2.31) | 1.28 (1.26, 1.31) |
| Diabetes |  |  |  |  |  |
| No History of Diabetes | Reference | Reference | Reference | Reference | Reference |
| History of Diabetes | 1.49 (1.43, 1.54) | 1.50 (1.45, 1.55) | 1.56 (1.53, 1.60) | 1.61 (1.56, 1.66) | 1.63 (1.60, 1.66) |
| Asthma |  |  |  |  |  |
| No History of Asthma | Reference | Reference | Reference | Reference | Reference |
| History of Asthma | 1.23 (1.18, 1.28) | 1.72 (1.67, 1.78) | 1.40 (1.37, 1.44) | 1.11 (1.08, 1.15) | 1.38 (1.35, 1.40) |
| Cancer |  |  |  |  |  |
| No History of Cancer | Reference | Reference | Reference | Reference | Reference |
| History of Cancer | 1.19 (1.14, 1.24) | 0.87 (0.84, 0.91) | 0.98 (0.96, 1.01) | 0.91 (0.88, 0.95) | 1.16 (1.13, 1.19) |
| PVD |  |  |  |  |  |
| No History of PVD | Reference | Reference | Reference | Reference | Reference |
| History of PVD | 1.02 (0.98, 1.08) | 1.34 (1.29, 1.39) | 1.13 (1.10, 1.16) | 0.98 (0.94, 1.01) | 1.18 (1.15, 1.21) |
| Liver Disease |  |  |  |  |  |
| No History of Liver Disease | Reference | Reference | Reference | Reference | Reference |
| History of Liver Disease | 1.93 (1.84, 2.03) | 1.53 (1.46, 1.60) | 1.60 (1.55, 1.65) | 1.73 (1.67, 1.80) | 1.66 (1.61, 1.70) |
| Obesity |  |  |  |  |  |
| No History of Obesity | Reference | Reference | Reference | Reference | Reference |
| History of Obesity | 1.45 (1.40, 1.50) | 1.41 (1.37, 1.46) | 1.39 (1.36, 1.42) | 1.39 (1.35, 1.42) | 1.45 (1.43, 1.47) |
| CAD |  |  |  |  |  |
| No History of CAD | Reference | N/A | N/A | Reference | Reference |
| History of CAD | 0.92 (0.88, 0.97) | N/A | N/A | 0.91 (0.88, 0.94) | 1.06 (1.03, 1.09) |
| CHF |  |  |  |  |  |
| No History of CHF | Reference | N/A | N/A | Reference | Reference |
| History of CHF | 1.84 (1.76, 1.92) | N/A | N/A | 1.72 (1.66, 1.78) | 2.05 (1.99, 2.10) |
| Vaccination Status Before COVID-19 | | |  |  |  |
| Non-Breakthrough Infection | Reference | Reference | Reference | Reference | Reference |
| VAX2 Breakthrough Infection | 0.32 (0.30, 0.34) | 0.50 (0.48, 0.52) | 0.41 (0.40, 0.42) | 0.36 (0.34, 0.37) | 0.40 (0.39, 0.40) |
| VAX3 Breakthrough Infection | 0.35 (0.28, 0.42) | 0.51 (0.43, 0.60) | 0.45 (0.40, 0.50) | 0.42 (0.36, 0.49) | 0.41 (0.37, 0.45) |

1. **Wave 4: Omicron (B.1.1.529, BA.2, BA.2.12.1)**

| **Characteristic** | **Death,**  **aHR (95% CI)** | **MACE,**  **aHR (95% CI)** | **MARCE,**  **aHR (95% CI)** | **AKI/Dialysis,**  **aHR (95% CI)** | **Hospitalization,**  **aOR (95% CI)** |
| --- | --- | --- | --- | --- | --- |
| Sex |  |  |  |  |  |
| Female | Reference | Reference | Reference | Reference | Reference |
| Male | 1.54 (1.49, 1.59) | 1.48 (1.44, 1.51) | 1.52 (1.50, 1.55) | 1.54 (1.51, 1.58) | 0.97 (0.96, 0.98) |
| Age at COVID-19 Diagnosis | 1.07 (1.07, 1.07) | 1.05 (1.05, 1.05) | 1.05 (1.05, 1.05) | 1.03 (1.03, 1.04) | 1.02 (1.02, 1.02) |
| Race/Ethnicity |  |  |  |  |  |
| White | Reference | Reference | Reference | Reference | Reference |
| Black or African American | 1.24 (1.18, 1.31) | 1.56 (1.51, 1.61) | 1.72 (1.68, 1.76) | 1.95 (1.89, 2.00) | 1.54 (1.52, 1.57) |
| Hispanic or Latino | 1.06 (1.00, 1.13) | 0.95 (0.91, 1.00) | 1.11 (1.08, 1.15) | 1.32 (1.27, 1.37) | 1.62 (1.59, 1.65) |
| Other/Unknown | 1.04 (0.98, 1.10) | 0.97 (0.93, 1.01) | 1.00 (0.98, 1.03) | 1.08 (1.04, 1.13) | 0.88 (0.86, 0.89) |
| SOT Status |  |  |  |  |  |
| Non-SOT Recipient | Reference | Reference | Reference | Reference | Reference |
| SOT Recipient | 1.58 (1.46, 1.70) | 1.38 (1.31, 1.46) | 1.90 (1.84, 1.96) | 2.07 (1.99, 2.15) | 1.60 (1.54, 1.66) |
| CKD |  |  |  |  |  |
| No History of CKD | Reference | Reference | Reference | Reference | Reference |
| History of CKD | 1.60 (1.53, 1.67) | 2.10 (2.04, 2.16) | 2.52 (2.47, 2.57) | 3.25 (3.15, 3.34) | 1.79 (1.75, 1.83) |
| Hypertension |  |  |  |  |  |
| No History of Hypertension | Reference | Reference | Reference | Reference | Reference |
| History of Hypertension | 1.26 (1.21, 1.32) | 3.13 (3.03, 3.24) | 2.46 (2.40, 2.52) | 2.48 (2.40, 2.57) | 1.40 (1.38, 1.43) |
| Diabetes |  |  |  |  |  |
| No History of Diabetes | Reference | Reference | Reference | Reference | Reference |
| History of Diabetes | 1.36 (1.31, 1.41) | 1.54 (1.50, 1.58) | 1.54 (1.51, 1.57) | 1.52 (1.49, 1.56) | 1.54 (1.52, 1.56) |
| Asthma |  |  |  |  |  |
| No History of Asthma | Reference | Reference | Reference | Reference | Reference |
| History of Asthma | 1.28 (1.23, 1.33) | 1.77 (1.73, 1.82) | 1.51 (1.48, 1.54) | 1.19 (1.16, 1.23) | 1.44 (1.41, 1.46) |
| Cancer |  |  |  |  |  |
| No History of Cancer | Reference | Reference | Reference | Reference | Reference |
| History of Cancer | 1.64 (1.58, 1.70) | 0.87 (0.84, 0.90) | 1.08 (1.06, 1.11) | 1.04 (1.01, 1.08) | 1.46 (1.43, 1.49) |
| PVD |  |  |  |  |  |
| No History of PVD | Reference | Reference | Reference | Reference | Reference |
| History of PVD | 1.05 (1.00, 1.09) | 1.35 (1.31, 1.39) | 1.21 (1.18, 1.24) | 0.99 (0.96, 1.02) | 1.25 (1.22, 1.27) |
| Liver Disease |  |  |  |  |  |
| No History of Liver Disease | Reference | Reference | Reference | Reference | Reference |
| History of Liver Disease | 2.19 (2.09, 2.29) | 1.38 (1.34, 1.43) | 1.55 (1.51, 1.59) | 1.64 (1.58, 1.69) | 1.64 (1.61, 1.68) |
| Obesity |  |  |  |  |  |
| No History of Obesity | Reference | Reference | Reference | Reference | Reference |
| History of Obesity | 0.96 (0.92, 0.99) | 1.25 (1.22, 1.28) | 1.09 (1.07, 1.11) | 1.01 (0.98, 1.03) | 1.09 (1.08, 1.11) |
| CAD |  |  |  |  |  |
| No History of CAD | Reference | N/A | N/A | Reference | Reference |
| History of CAD | 0.95 (0.91, 0.99) | N/A | N/A | 1.00 (0.97, 1.03) | 1.27 (1.24, 1.30) |
| CHF |  |  |  |  |  |
| No History of CHF | Reference | N/A | N/A | Reference | Reference |
| History of CHF | 2.15 (2.06, 2.25) | N/A | N/A | 1.94 (1.89, 2.00) | 2.42 (2.37, 2.47) |
| Vaccination Status Before COVID-19 | | |  |  |  |
| Non-Breakthrough Infection | Reference | Reference | Reference | Reference | Reference |
| VAX2 Breakthrough Infection | 0.55 (0.52, 0.58) | 0.64 (0.62, 0.66) | 0.58 (0.57, 0.60) | 0.53 (0.51, 0.55) | 0.55 (0.54, 0.56) |
| VAX3 Breakthrough Infection | 0.33 (0.31, 0.35) | 0.47 (0.45, 0.49) | 0.41 (0.40, 0.42) | 0.37 (0.35, 0.38) | 0.40 (0.40, 0.41) |

1. **Wave 5: Omicron (BA.5, BQ.1.1, XBB.1.5)**

| **Characteristic** | **Death,**  **aHR (95% CI)** | **MACE,**  **aHR (95% CI)** | **MARCE,**  **aHR (95% CI)** | **AKI/Dialysis,**  **aHR (95% CI)** | **Hospitalization,**  **aOR (95% CI)** |
| --- | --- | --- | --- | --- | --- |
| Sex |  |  |  |  |  |
| Female | Reference | Reference | Reference | Reference | Reference |
| Male | 1.34 (1.29, 1.39) | 1.40 (1.37, 1.43) | 1.44 (1.41, 1.46) | 1.44 (1.40, 1.47) | 0.98 (0.96, 0.99) |
| Age at COVID-19 Diagnosis | 1.06 (1.06, 1.06) | 1.05 (1.05, 1.05) | 1.04 (1.04, 1.04) | 1.03 (1.03, 1.03) | 1.01 (1.01, 1.01) |
| Race/Ethnicity |  |  |  |  |  |
| White | Reference | Reference | Reference | Reference | Reference |
| Black or African American | 1.18 (1.11, 1.26) | 1.39 (1.35, 1.44) | 1.51 (1.47, 1.54) | 1.68 (1.62, 1.74) | 1.29 (1.27, 1.32) |
| Hispanic or Latino | 0.91 (0.84, 0.99) | 1.02 (0.97, 1.07) | 1.08 (1.04, 1.12) | 1.23 (1.18, 1.29) | 1.40 (1.37, 1.43) |
| Other/Unknown | 0.97 (0.91, 1.03) | 0.96 (0.93, 1.01) | 0.97 (0.94, 1.00) | 1.05 (1.00, 1.09) | 0.83 (0.81, 0.85) |
| SOT Status |  |  |  |  |  |
| Non-SOT Recipient | Reference | Reference | Reference | Reference | Reference |
| SOT Recipient | 1.28 (1.16, 1.41) | 1.31 (1.24, 1.39) | 1.81 (1.75, 1.88) | 1.94 (1.86, 2.03) | 1.55 (1.49, 1.62) |
| CKD |  |  |  |  |  |
| No History of CKD | Reference | Reference | Reference | Reference | Reference |
| History of CKD | 1.54 (1.48, 1.61) | 1.99 (1.94, 2.05) | 2.33 (2.29, 2.38) | 3.07 (2.98, 3.16) | 1.62 (1.59, 1.66) |
| Hypertension |  |  |  |  |  |
| No History of Hypertension | Reference | Reference | Reference | Reference | Reference |
| History of Hypertension | 1.04 (0.99, 1.09) | 2.71 (2.62, 2.81) | 2.16 (2.11, 2.21) | 2.13 (2.05, 2.21) | 1.36 (1.34, 1.38) |
| Diabetes |  |  |  |  |  |
| No History of Diabetes | Reference | Reference | Reference | Reference | Reference |
| History of Diabetes | 1.25 (1.20, 1.30) | 1.47 (1.44, 1.51) | 1.48 (1.45, 1.50) | 1.47 (1.43, 1.52) | 1.43 (1.41, 1.45) |
| Asthma |  |  |  |  |  |
| No History of Asthma | Reference | Reference | Reference | Reference | Reference |
| History of Asthma | 1.29 (1.23, 1.34) | 1.70 (1.66, 1.75) | 1.50 (1.47, 1.53) | 1.16 (1.12, 1.19) | 1.39 (1.37, 1.42) |
| Cancer |  |  |  |  |  |
| No History of Cancer | Reference | Reference | Reference | Reference | Reference |
| History of Cancer | 1.91 (1.84, 1.99) | 0.88 (0.86, 0.91) | 1.10 (1.07, 1.12) | 1.06 (1.03, 1.10) | 1.39 (1.37, 1.42) |
| PVD |  |  |  |  |  |
| No History of PVD | Reference | Reference | Reference | Reference | Reference |
| History of PVD | 1.16 (1.11, 1.22) | 1.42 (1.38, 1.46) | 1.30 (1.27, 1.33) | 1.05 (1.01, 1.08) | 1.28 (1.25, 1.30) |
| Liver Disease |  |  |  |  |  |
| No History of Liver Disease | Reference | Reference | Reference | Reference | Reference |
| History of Liver Disease | 2.20 (2.09, 2.32) | 1.34 (1.29, 1.38) | 1.53 (1.49, 1.57) | 1.69 (1.64, 1.75) | 1.60 (1.56, 1.63) |
| Obesity |  |  |  |  |  |
| No History of Obesity | Reference | Reference | Reference | Reference | Reference |
| History of Obesity | 0.73 (0.70, 0.76) | 1.18 (1.15, 1.21) | 0.99 (0.97, 1.01) | 0.86 (0.84, 0.89) | 0.90 (0.89, 0.92) |
| CAD |  |  |  |  |  |
| No History of CAD | Reference | N/A | N/A | Reference | Reference |
| History of CAD | 0.98 (0.94, 1.03) | N/A | N/A | 1.05 (1.02, 1.08) | 1.29 (1.27, 1.32) |
| CHF |  |  |  |  |  |
| No History of CHF | Reference | N/A | N/A | Reference | Reference |
| History of CHF | 2.54 (2.42, 2.65) | N/A | N/A | 2.07 (2.01, 2.14) | 2.56 (2.51, 2.61) |
| Vaccination Status Before COVID-19 | | |  |  |  |
| Non-Breakthrough Infection | Reference | Reference | Reference | Reference | Reference |
| VAX2 Breakthrough Infection | 0.70 (0.66, 0.74) | 0.68 (0.66, 0.71) | 0.67 (0.66, 0.69) | 0.63 (0.60, 0.65) | 0.68 (0.66, 0.69) |
| VAX3 Breakthrough Infection | 0.55 (0.53, 0.58) | 0.54 (0.53, 0.56) | 0.52 (0.51, 0.53) | 0.47 (0.45, 0.48) | 0.48 (0.47, 0.49) |

**Supplemental Table 3: Crude Event Rates For Non-Immunosuppressed/Immunocompromised and Solid Organ Transplant Populations by Organ Type Over Waves of the Pandemic.**

| **Characteristic** | **Wave 1**  **Ancestral**  **COVID-19** | **Wave 2**  **Alpha (B.1.1.7),**  **Beta (B.1.351),**  **Gamma (P.1)** | **Wave 3**  **Delta**  **(B.1.617.2)** | **Wave 4**  **Omicron (B.1.1.529, BA.2, BA.2.12.1)** | **Wave 5**  **Omicron**  **(BA.5, BQ.1.1, XBB.1.5)** |
| --- | --- | --- | --- | --- | --- |
| **Mortality** | | | | | |
| Non-ISC | 25,736 / 1,481,743 (1.7%) | 10,327 / 649,065 (1.6%) | 15,127 / 918,803 (1.6%) | 13,811 / 1,504,310 (0.92%) | 11,294 / 915,261 (1.2%) |
| **Kidney** | 515 / 6,808 (7.6%) | 284 / 4,112 (6.9%) | 469 / 5,073 (9.2%) | 547 / 10,565 (5.2%) | 261 / 6,854 (3.8%) |
| **Liver** | 119 / 1,591 (7.5%) | 50 / 949 (5.3%) | 87 / 1,153 (7.5%) | 116 / 2,703 (4.3%) | 68 / 2,149 (3.2%) |
| **Lung** | 83 / 852 (9.7%) | 44 / 550 (8.0%) | 72 / 695 (10%) | 101 / 1,522 (6.6%) | 72 / 1,264 (5.7%) |
| **Heart** | 68 / 1,246 (5.5%) | 53 / 724 (7.3%) | 68 / 840 (8.1%) | 70 / 1,682 (4.2%) | 48 / 1,298 (3.7%) |
| **MARCE** | | | | | |
| Non-ISC | 67,034 / 1,481,743 (4.5%) | 33,671 / 649,065 (5.2%) | 41,910 / 918,803 (4.6%) | 53,706 / 1,504,310 (3.6%) | 28,179 / 915,261 (3.1%) |
| **Kidney** | 2,551 / 6,808 (37%) | 1,488 / 4,112 (36%) | 1,788 / 5,073 (35%) | 3,180 / 10,565 (30%) | 744 / 6,854 (11%) |
| **Liver** | 431 / 1,591 (27%) | 235 / 949 (25%) | 311 / 1,153 (27%) | 620 / 2,703 (23%) | 136 / 2,149 (6.3%) |
| **Lung** | 268 / 852 (31%) | 185 / 550 (34%) | 249 / 695 (36%) | 453 / 1,522 (30%) | 126 / 1,264 (10%) |
| **Heart** | 515 / 1,246 (41%) | 310 / 724 (43%) | 349 / 840 (42%) | 613 / 1,682 (36%) | 298 / 1,298 (23%) |
| **MACE** | | | | | |
| Non-ISC | 27,117 / 1,481,743 (1.8%) | 15,097 / 649,065 (2.3%) | 17,442 / 918,803 (1.9%) | 27,701 / 1,504,310 (1.8%) | 50,600 / 915,261 (5.5%) |
| **Kidney** | 863 / 6,808 (13%) | 484 / 4,112 (12%) | 553 / 5,073 (11%) | 1,100 / 10,565 (10%) | 2,007 / 6,854 (29%) |
| **Liver** | 116 / 1,591 (7.3%) | 69 / 949 (7.3%) | 93 / 1,153 (8.1%) | 179 / 2,703 (6.6%) | 494 / 2,149 (23%) |
| **Lung** | 91 / 852 (11%) | 76 / 550 (14%) | 88 / 695 (13%) | 157 / 1,522 (10%) | 353 / 1,264 (28%) |
| **Heart** | 308 / 1,246 (25%) | 197 / 724 (27%) | 197 / 840 (23%) | 389 / 1,682 (23%) | 447 / 1,298 (34%) |
| **AKI** | | | | | |
| Non-ISC | 37,242 / 1,481,743 (2.5%) | 19,240 / 649,065 (3.0%) | 23,744 / 918,803 (2.6%) | 27,504 / 1,504,310 (1.8%) | 24,253 / 915,261 (2.6%) |
| **Kidney** | 2,085 / 6,808 (31%) | 1,217 / 4,112 (30%) | 1,482 / 5,073 (29%) | 2,551 / 10,565 (24%) | 1,558 / 6,854 (23%) |
| **Liver** | 335 / 1,591 (21%) | 185 / 949 (19%) | 241 / 1,153 (21%) | 484 / 2,703 (18%) | 410 / 2,149 (19%) |
| **Lung** | 211 / 852 (25%) | 143 / 550 (26%) | 185 / 695 (27%) | 368 / 1,522 (24%) | 257 / 1,264 (20%) |
| **Heart** | 322 / 1,246 (26%) | 194 / 724 (27%) | 224 / 840 (27%) | 369 / 1,682 (22%) | 252 / 1,298 (19%) |
| **Hospitalization** | | | | | |
| Non-ISC | 167,520 / 1,481,743 (11%) | 86,809 / 649,065 (13%) | 108,909 / 918,803 (12%) | 134,694 / 1,504,310 (9.0%) | 120,010 / 915,261 (13%) |
| **Kidney** | 3,302 / 6,808 (49%) | 1,845 / 4,112 (45%) | 2,088 / 5,073 (41%) | 3,798 / 10,565 (36%) | 2,621 / 6,854 (38%) |
| **Liver** | 708 / 1,591 (45%) | 413 / 949 (44%) | 447 / 1,153 (39%) | 948 / 2,703 (35%) | 813 / 2,149 (38%) |
| **Lung** | 437 / 852 (51%) | 286 / 550 (52%) | 340 / 695 (49%) | 644 / 1,522 (42%) | 559 / 1,264 (44%) |
| **Heart** | 627 / 1,246 (50%) | 368 / 724 (51%) | 385 / 840 (46%) | 685 / 1,682 (41%) | 555 / 1,298 (43%) |

**Supplemental Table 4: Adjusted Risk for Solid Organ Transplant Recipients by Organ Type Relative to the Non-Immunosuppressed/ Immunocompromised Population Over Waves of the Pandemic.**

| **Characteristic** | **Wave 1**  **Ancestral**  **COVID-19** | **Wave 2**  **Alpha (B.1.1.7),**  **Beta (B.1.351),**  **Gamma (P.1)** | **Wave 3**  **Delta**  **(B.1.617.2)** | **Wave 4**  **Omicron (B.1.1.529, BA.2, BA.2.12.1)** | **Wave 5**  **Omicron**  **(BA.5, BQ.1.1, XBB.1.5)** |
| --- | --- | --- | --- | --- | --- |
| **Mortality (HR)** | | | | | |
| **Kidney** | 1.50 (1.37, 1.64) | 1.61 (1.43, 1.83) | 1.83 (1.66, 2.02) | 1.84 (1.68, 2.01) | 1.46 (1.28, 1.66) |
| **Liver** | 1.16 (0.97, 1.40) | 0.82 (0.62, 1.08) | 1.15 (0.92, 1.42) | 1.06 (0.88, 1.28) | 0.83 (0.65, 1.06) |
| **Lung** | 2.06 (1.66, 2.56) | 2.07 (1.54, 2.79) | 1.93 (1.53, 2.43) | 2.27 (1.86, 2.76) | 1.97 (1.56, 2.49) |
| **Heart** | 0.71 (0.56, 0.90) | 1.13 (0.86, 1.48) | 1.08 (0.85, 1.37) | 0.89 (0.70, 1.13) | 0.87 (0.65, 1.16) |
| **MARCE (HR)** | | | | | |
| **Kidney** | 1.92 (1.84, 2.01) | 1.70 (1.61, 1.80) | 1.77 (1.69, 1.87) | 1.87 (1.80, 1.95) | 1.83 (1.75, 1.92) |
| **Liver** | 1.56 (1.41, 1.72) | 1.22 (1.07, 1.39) | 1.50 (1.34, 1.69) | 1.57 (1.44, 1.70) | 1.47 (1.34, 1.61) |
| **Lung** | 2.04 (1.81, 2.30) | 2.27 (1.97, 2.63) | 2.20 (1.94, 2.49) | 2.20 (2.00, 2.41) | 1.85 (1.66, 2.05) |
| **Heart** | 2.05 (1.88, 2.24) | 2.15 (1.92, 2.41) | 2.29 (2.06, 2.54) | 2.28 (2.10, 2.47) | 2.23 (2.03, 2.45) |
| **MACE (HR)** | | | | | |
| **Kidney** | 1.36 (1.27, 1.46) | 1.18 (1.07, 1.30) | 1.20 (1.10, 1.31) | 1.25 (1.18, 1.34) | 1.22 (1.13, 1.32) |
| **Liver** | 0.96 (0.79, 1.15) | 0.84 (0.66, 1.07) | 1.05 (0.85, 1.29) | 0.96 (0.82, 1.11) | 0.82 (0.69, 0.97) |
| **Lung** | 1.41 (1.14, 1.73) | 1.78 (1.42, 2.23) | 1.51 (1.23, 1.87) | 1.33 (1.14, 1.56) | 1.13 (0.95, 1.35) |
| **Heart** | 2.60 (2.32, 2.92) | 2.91 (2.52, 3.36) | 2.68 (2.32, 3.09) | 2.77 (2.51, 3.07) | 2.72 (2.43, 3.06) |
| **AKI (HR)** | | | | | |
| **Kidney** | 2.00 (1.90, 2.09) | 1.81 (1.70, 1.93) | 1.88 (1.78, 2.00) | 2.10 (2.00, 2.19) | 2.02 (1.91, 2.14) |
| **Liver** | 1.92 (1.72, 2.15) | 1.42 (1.23, 1.65) | 1.78 (1.56, 2.03) | 2.05 (1.87, 2.25) | 2.07 (1.87, 2.29) |
| **Lung** | 2.75 (2.40, 3.15) | 2.81 (2.38, 3.31) | 2.58 (2.23, 2.98) | 3.05 (2.75, 3.39) | 2.25 (1.99, 2.55) |
| **Heart** | 1.41 (1.26, 1.57) | 1.37 (1.19, 1.59) | 1.53 (1.34, 1.75) | 1.45 (1.31, 1.61) | 1.31 (1.16, 1.49) |
| **Hospitalization (OR)** | | | | | |
| **Kidney** | 1.81 (1.71, 1.92) | 1.44 (1.34, 1.55) | 1.60 (1.50, 1.71) | 1.53 (1.46, 1.61) | 1.54 (1.45, 1.62) |
| **Liver** | 1.78 (1.59, 2.00) | 1.47 (1.27, 1.70) | 1.57 (1.37, 1.79) | 1.73 (1.58, 1.89) | 1.65 (1.50, 1.82) |
| **Lung** | 2.52 (2.17, 2.93) | 2.83 (2.35, 3.41) | 2.71 (2.30, 3.20) | 2.48 (2.21, 2.77) | 2.08 (1.84, 2.34) |
| **Heart** | 1.46 (1.28, 1.66) | 1.38 (1.17, 1.62) | 1.44 (1.24, 1.68) | 1.21 (1.09, 1.35) | 1.14 (1.01, 1.28) |

*Adjusted for* *sex, age, race/ethnicity (White, Black, Hispanic or Latino, Other), comorbidities (chronic kidney disease, hypertension, diabetes, asthma/chronic obstructive pulmonary disease, cancer, peripheral vascular disease, liver disease, obesity, coronary artery disease, congestive heart failure), and vaccination status (no complete vaccination series documented, or breakthrough infection (VAX2: being ≥14 days post two doses for mRNA vaccines, one dose for Johnson & Johnson/Janssen vaccine, or two doses for other vaccines; VAX3: being ≥14 days post a booster dose of any of the above vaccine preparations following VAX2))*


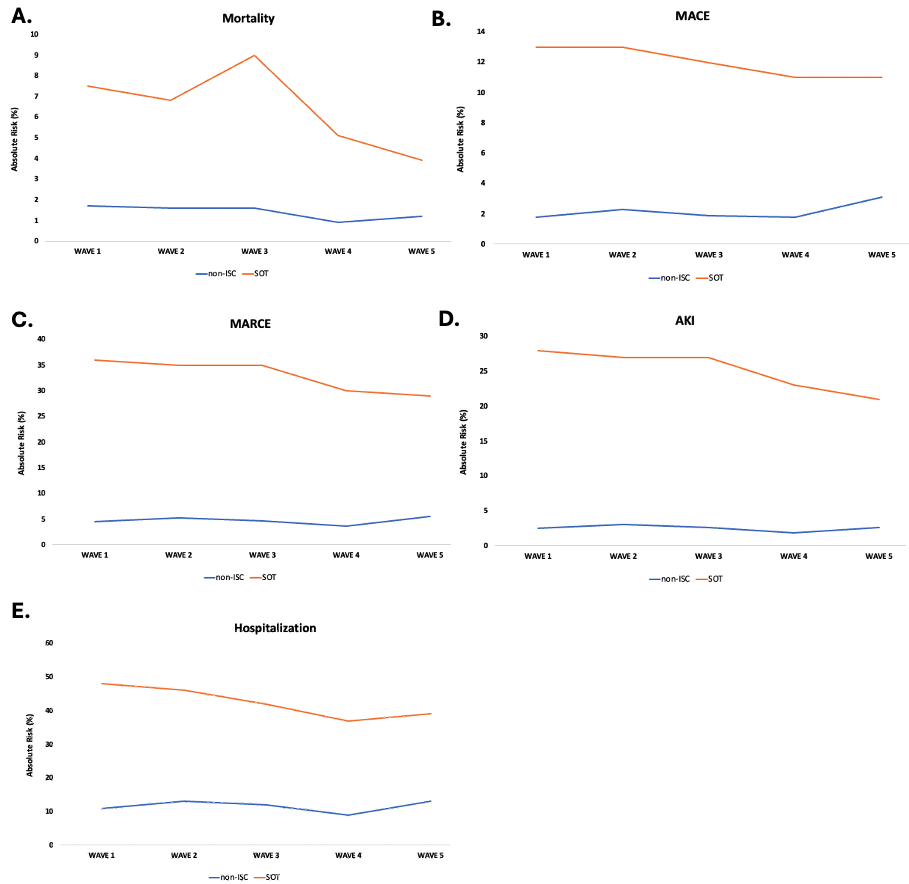


**Supplemental Figure 1: Absolute Risk of A. mortality, B. major adverse cardiac event (MACE), C. major adverse renal or cardiac event (MARCE), D. acute kidney injury (AKI), or E. hospitalization in the 90 days after COVID-19 diagnosis in solid organ transplant (SOT) and non-immunosuppressed/immunocompromised (non-ISC) patients.** *Wave 1: Ancestral COVID; 01/01/2020-12/31/2020; Wave 2: Alpha; 01/01/2021-06/25/2021; Wave 3: Delta; 06/26/2021-12/17/2021; Wave 4: Omicron; 12/18/2021-07/01/2022; Wave 5: Omicron; 07/02/2022-03/31/2023*
